# Genome-wide CNV analysis in mouse induced pluripotent stem cells reveals dosage effect of pluripotent factors on genome integrity

**DOI:** 10.1186/1471-2164-15-79

**Published:** 2014-01-28

**Authors:** Yulin Chen, Lin Guo, Jiekai Chen, Xiangjie Zhao, Weichen Zhou, Cheng Zhang, Jiucun Wang, Li Jin, Duanqing Pei, Feng Zhang

**Affiliations:** 1 State Key Laboratory of Genetic Engineering and Ministry of Education Key Laboratory of Contemporary Anthropology, School of Life Sciences, Fudan University, Shanghai 200433, China; 2 Key Laboratory of Regenerative Biology and Guangdong Provincial Key Laboratory of Stem Cell and Regenerative Medicine, South China Institute for Stem Cell Biology and Regenerative Medicine, Guangzhou Institutes of Biomedicine and Health, Chinese Academy of Sciences, Guangzhou 510530, China; 3 Collaborative Innovation Center for Genetics and Development, School of Life Sciences, Fudan University, Shanghai 200433, China

**Keywords:** CNV, Genome integrity, Induced pluripotent stem cell, Reprogramming factor, Reprogramming kinetics

## Abstract

**Background:**

Induced pluripotent stem cells (iPSCs) derived from somatic cells have enormous potential for clinical applications. Notably, it was recently reported that reprogramming from somatic cells to iPSCs can induce genomic copy number variation (CNV), which is one of the major genetic causes of human diseases. However it was unclear if this genome instability is dependent on reprogramming methods and/or the genetic background of donor cells. Furthermore, genome-wide CNV analysis is technically challenging and CNV data need to be interpreted with care.

**Results:**

In order to carefully investigate the possible CNV instability during somatic reprogramming, we performed genome-wide CNV analyses with 41 mouse iPSC lines generated from the same parental donor; therefore, the donor’s genetic background can be controlled. Different reprogramming factor combinations and dosages were used for investigating potential method-dependent effects on genome integrity. We detected 63 iPSC CNVs using high-resolution comparative genomic hybridization. Intriguingly, CNV rates were negatively associated with the dosages of classic factor(s). Furthermore, the use of high-performance engineered factors led to less CNVs than the classic factor(s) of the same dosage.

**Conclusion:**

Our observations suggest that sufficient reprogramming force can protect the genome from CNV instability during the reprogramming process.

## Background

Induced pluripotent stem cells (iPSCs), which are derived from somatic cells through reprogramming via several methods [1], have enormous numbers of potential applications, particularly in regenerative medicine, disease modeling and drug screening [2]. However, safe and effective reprogramming methods remain to be described for producing high-quality iPSCs [3]. Before developing personalized stem cell therapies, genome integrity and other safety concerns of iPSC technology must be addressed [4], particularly as genome stability can have profound effects on pluripotency, differentiation and the tumorigenicity of resulting iPSCs [5]. Notably, it was recently shown that the process of reprogramming somatic cells to iPSCs could induce genome alterations such as copy number variation (CNV) [6-8]. Current evidence suggests that these reprogramming-associated CNVs could be either *de novo* mutations or enriched mosaic variations in donor cells [9,10]. CNVs are one of the major genetic causes of human diseases [11]; therefore, it is imperative to carefully investigate possible CNV instability during somatic reprogramming before using iPSCs in a clinical or therapeutic setting.

Genome-wide CNV analysis is technically challenging and the CNV data need to be interpreted with caution [11,12]. To date, several genome technologies have been utilized for genomic CNV analysis [12]; however, the following points need to be taken into consideration before confirming CNV instability in iPSCs. Firstly, technical limitations exist for identifying CNVs accurately, as the CNV calls of SNP microarrays (SNP for single nucleotide polymorphism) are highly dependent on the external reference set used for the analysis [12]. The lack of internal references on SNP microarrays can lead to low signal-to-noise ratios in the process of CNV calling whilst CNV data obtained by comparative genomic hybridization (CGH) technology are more reliable [12]. Secondly, previous CNV calls in iPSCs cannot readily distinguish between reprogramming-associated CNVs (either *de novo* CNV or selected mosaic CNV) [10,13] and pre-existing germ-line CNVs in parental cells. Finally, it is still unknown whether the reported CNV instability is dependent on reprogramming methods or due to the genetic backgrounds of parental cells [6,14], which can potentially cause method-specific or donor-specific genome instability.

**Figure 1:**
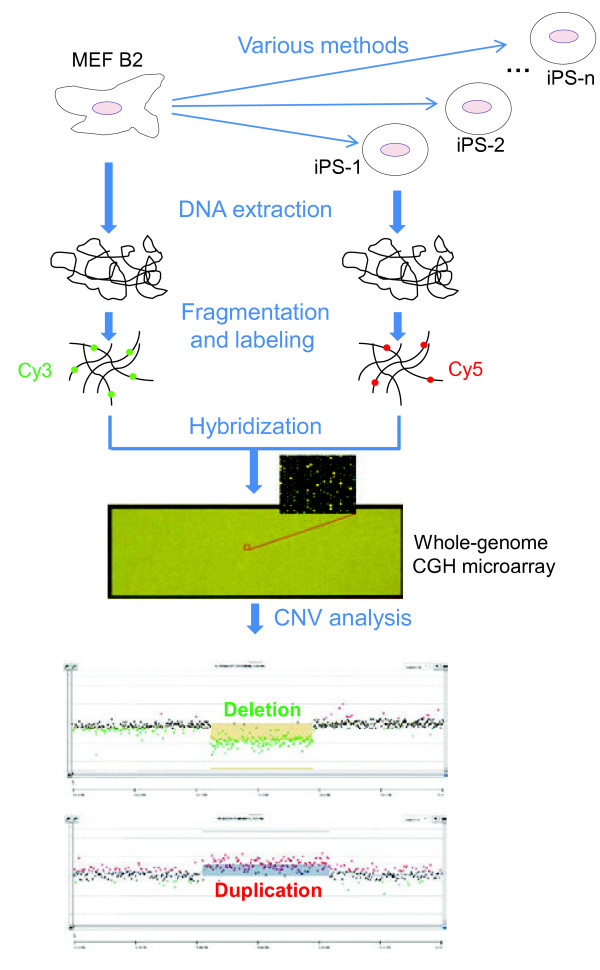
**The experimental flow of genome-wide CNV analyses in miPSCs using high-density CGH microarrays.** A total of 41 miPSC lines were derived from the same donor of MEF B2 using different reprogramming factors and/or dosages. Genomic DNAs of the parental MEF B2 and various miPSC lines were respectively extracted and fragmented. Cy5-dUTP was used for labeling miPSC DNAs and Cy3-dUTP for the donor DNAs. Each labeled miPSC DNA was hybridized together with a labeled donor DNA onto the mouse genome CGH microarrays. Microarray handling and data analysis were conducted following the Agilent oligonucleotide CGH protocol. Examples were shown for a copy number loss (deletion; depicted by green concave) and a copy number gain (duplication; depicted by red convex) at the bottom.

Considering the above concerns, we addressed the issue of CNV instability in iPSCs by taking into account the following factors in our study design. Mouse iPSCs (miPSCs) were generated from the same parental donor to exclude the effect of the genetic background. Various combinations of reprogramming factors and dosages were used for CNV comparison between reprogramming methods. In addition, a high-density CGH microarray assay comparing miPSCs with their parental donor cells was used for genome-wide screening for the CNVs associated with cell reprogramming (Figure 1). Intriguingly, our observations revealed the dosage effect of pluripotent factors on genome integrity during somatic reprogramming.

## Results

Initially, we obtained 16 miPSC lines with the three “Yamanaka” factors (Oct4/Klf4/Sox2, OKS) [15,16] and single Oct4 [17] (Additional file 1): eight miPSC lines obtained by O_0.5, the other eight lines obtained by OKS_1.5. The dosage of each factor in these two methods was equivalent. Intriguingly, we identified 24 CNVs in eight miPSC lines of O_0.5 (i.e. 3.0 CNV/miPSC) and seven CNVs in eight lines of OKS_1.5 (i.e. approximately 0.9 CNV/miPSC) (Additional file 2). The rates of miPSC CNVs between these two reprogramming methods are obviously different, suggesting that the strength of the iPSC reprogramming has an effect on genome integrity. Potentially this suggests that reduced diversity of reprogramming factors and/or reduced reprogramming dosages may induce more CNVs during somatic reprogramming.

**Figure 2:**
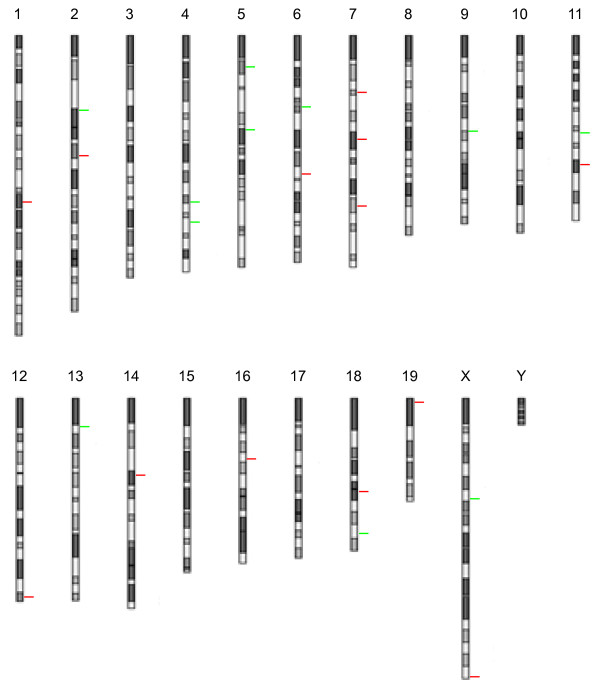
**The genome distribution of 24 loci of the miPSC CNVs identified in this study was shown.** The green bars indicate the deletion loci, and the red bars represent duplication loci.

To further investigate the possible roles of reprogramming factor diversity and/or dosage in CNV instability, we generated another three sets of miPSC lines from the same donor but using different factor combinations and low/high (i.e., 0.5 ml/1.5 ml) dosages (see Methods for details) (Additional file 1): five miPSC lines obtained by O_1.5, ten lines obtained by OKS_0.5, and ten lines obtained by engineered factors XYZK_0.5 [18]. Using CGH assay we compared the resulting miPSC genomes with their parental genomes and identified zero CNVs in five miPSC lines of O_1.5 (i.e. 0 CNV/miPSC), 25 CNVs in ten OKS_0.5 lines (i.e. 2.5 CNV/miPSC), and seven CNVs in ten XYZK_0.5 lines (i.e. 0.7 CNV/miPSC) (Additional file 2). In total, we screened the genomes of 41 miPSC lines and identified 63 CNVs across 24 genomic loci of the mouse genome (Figure 2 and Additional file 2).

**Figure 3:**
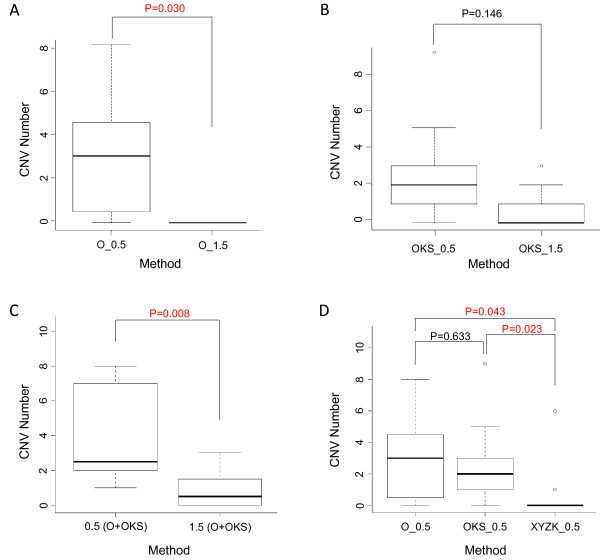
**Comparison of CNV rates between the miPSCs induced by different reprogramming methods. (A)** The comparison of CNV numbers between O_0.5 and O_1.5 miPSC lines. This difference is statistically significant (p-value = 0.030, Mann–Whitney *U* test with the exact significance). **(B)** The comparison of CNV numbers between OKS_0.5 and OKS_1.5 miPSC lines (Mann–Whitney *U* test with the exact significance). **(C)** The combinational analysis of **(A)** and **(B)**. A significant difference of CNV numbers between high and low reprogramming factor dosages was found (p-value = 0.008, ANOVA test for two-factor analysis). **(D)** The comparison of CNV numbers between the methods using diverse reprogramming factors with balanced dosages (Mann–Whitney *U* test with the exact significance). The CNVs are significantly less in the miPSCs of XYZK_0.5 than in those of O_0.5 (p-value = 0.043) and OKS_0.5 (p-value = 0.023).

To investigate the potential mechanism involved in genome instability of miPSCs, we compared the CNV rates between the various methods using the same reprogramming factor combinations with altered dosages. The Mann–Whitney *U* test with the exact significance was used for single-factor test and the ANOVA test was used for two-factor test. By comparing the CNV numbers between O_0.5 and O_1.5 miPSC lines, we observed more CNVs in O_0.5 miPSCs (24 CNVs/8 miPSCs) than in O_1.5 miPSCs (0 CNV/5 miPSCs). This difference is statistically significant (p-value = 0.030, Mann–Whitney *U* test) (Figure 3A). Similarly comparing the number of CNVs in OKS_0.5 (25 CNVs/10 miPSCs) and OKS _1.5 miPSC lines (7 CNVs/8 miPSCs), although not significant (p-value = 0.146, Mann–Whitney *U* test; Figure 3B) does still suggest that a low dosage of reprogramming factors may induce more CNVs than a high dosage. We also combined the CNV data in Figure 3A and 3B together based on their dosages. In the low dosage group (0.5 ml), 49 CNVs were detected in 18 miPSC lines (i.e. approximately 2.7 CNV/miPSC); while in the high dosage group (1.5 ml), eight CNVs were detected in 13 miPSC lines (i.e. approximately 0.6 CNV/miPSC). A significant difference in CNV rates was observed (p-value = 0.008, ANOVA test) (Figure 3C), which strongly supports that the dose of reprograming factors and consequently the reprogramming force can significantly affect the genome instability during reprogramming, with higher doses and stronger reprogramming providing a protective effect. Notably, recent studies have reported that reprogramming factor dosage can affect the epigenetic properties of iPSCs [3] and increased levels of Oct4 and Klf4 were observed to give rise to high-quality iPSCs [19,20].

To further explore the roles of reprogramming force in CNV instability of miPSCs, we compared CNV rates between diverse factor-combinations, while their total dosages remained the same. We introduced the engineered factors XYZK (Oct4-VP16, Sox2-VP16, Klf4 and Nanog-VP16) due to their strong promoting capability during reprogramming [18]. The CNV rates are: O_0.5 (24 CNVs/8 miPSCs), OKS_0.5 (25 CNVs/10 miPSCs) and XYZK_0.5 (7 CNVs/10 miPSCs) (Additional file 2). There is no significant difference between O_0.5 and OKS_0.5 (p-value = 0.633), however, the CNVs of XYZK_0.5 are significantly less than those in O_0.5 (p-value = 0.043) and those in OKS_0.5 (p-value = 0.023) (Figure 3D). Particularly important was the observation that all the seven CNVs detected in XYZK_0.5 came from just two of the ten iPSC lines and six of those seven CNVs were in a single iPSC line. The remaining eight iPSC lines of XYZK_0.5 ad zero CNVs (Additional file 2). This suggests that high-performance engineered factors XYZK are likely to help maintain the genome integrity by reducing reprogramming barriers. Consistently, these observations also support that sufficient reprogramming force has a positive role in iPSC genome integrity.

## Discussion

Mouse iPSCs were first generated by retroviral transduction of four transcription factors: Oct3/4, Sox2, Klf4 and c-Myc [1]. However, reactivation of c-Myc increases tumorigenicity in the chimeras, hindering clinical applications [21]. It was observed that the mice derived from c-Myc-free iPSCs showed a significantly reduced incidence of tumorigenicity compared with those derived by the four classic factors [16]. For the sake of high-quality iPSCs generation, we excluded c-Myc in our study design. Considering the low-efficiency of iPSCs induction without c-Myc, we also utilized the optimized reprogramming culture conditions with ultra-high efficiency on iPSCs generation [15].

Based on the reliable CGH technology, we found that reprogramming factor dosage is negatively associated with CNV rate. This result showed the possibility that sufficient reprogramming force may help maintain genome integrity during somatic reprogramming.

Since the reprogramming process is an artificial process that reverses the somatic cell fate into a pluripotent state, reprogramming faces various epigenetic barriers that were set during normal differentiation [3]. Previous evidence showed that the reprogramming process can broadly be divided into two phases: a long stochastic phase of gene activation and a shorter, hierarchical, more deterministic phase of gene activation [3]. The stochastic nature of the reprogramming process suggests that not genetic but epigenetic barriers can be seen as roadblocks in the journey to pluripotency [22]. Reprogramming factors initiate transcriptional effect as well as epigenetic regulation to help re-establishing pluripotency [23,24]. Moreover, some regulators or chemicals, such as Jhdm1b, valporic acid and vitamin C, can overcome these epigenetic barriers and so markedly enhance reprogramming [3,25,26]. These observations suggest that the strength of reprogramming targeting epigenetic barriers is important for successful reprogramming. On the other hand, the iPSCs derived from the stochastic reprogramming phase represent the cells experiencing greater epigenetic changes from the somatic state to a pluripotent one, which could be recognized as a kind of pressure. CNV instability investigated in this study may serve as pressure-induced factors that take part in overcoming epigenetic roadblocks. Therefore, we suggest that iPSCs might experience more genome instability during the reprogramming process if the strength of reprogramming is not enough. Conversely, sufficient reprogramming force will lead to much fewer CNVs. Nevertheless, this hypothesis should be investigated further.

In total we performed genome-wide CNV analyses on 41 miPSC lines derived by different reprogramming factors and/or dosages and detected 63 miPSC CNVs. The average CNV rate is approximately 1.5 per miPSC line, which suggests that the CNVs associated with cell reprogramming is not frequent. The choices of appropriate reprogramming methods with sufficient reprogramming force are likely to help maintain genome integrity of iPSCs.

## Conclusions

In summary, we showed, using the CGH microarray assay to directly compare the CNV status of miPSCs to their parental cells is reliable to identify CNV alterations associated with cell reprogramming. Based on the genome-wide analyses of 41 miPSC lines derived by different methods, we suggest that increasing factor dosages, or using high-performance engineered factors [18], is beneficial for the genome integrity of the resulting miPSCs. Our observations highlight the importance of further investigations on the mechanisms and kinetics of cell reprogramming and their effects on iPSC genome integrity.

## Methods

### Mouse iPSCs generated from the same donor

An embryonic fibroblast cell line (MEF B2) derived from the OG2 mouse was used as the parental donor. The donor cells were infected with retroviruses carrying the indicated reprogramming factors for two days, and then were cultured in iCD1 medium for the generation of iPSCs [15]. We normalized the virus with equal titer (low dosage, MOI = 15 when 0.5 ml virus was used; high dosage, MOI = 45 when 1.5 ml virus was used) according to the titer detecting by Takara Retrovirus Titer Set. In total, we obtained eight miPSC lines using single-factor Oct4 (0.5 ml Oct4, i.e. O_0.5), five lines using single-factor Oct4 (1.5 ml Oct4, i.e. O_1.5), ten lines using three-factor combination (0.167 ml Oct4, 0.167 ml Klf4, and 0.167 ml Sox2, i.e. OKS_0.5), eight lines using three-factor combination (0.5 ml Oct4, 0.5 ml Klf4, and 0.5 ml Sox2, i.e. OKS_1.5), and ten lines obtained by previously reported engineered factors (0.125 ml Oct4-VP16 (X), 0.125 ml Sox2-VP16 (Y), 0.125 ml Nanog-VP16 (Z) and 0.125 ml Klf4, i.e. XYZK_0.5) [18]. The reprogramming efficiencies of different factor combinations were described in previous studies [15,17,18]. The iPSC colonies were picked based on Oct4-GFP expression and were validated with a normal karyotype. All of the 41 miPSC lines were harvested at passage 4 for further analysis. All the miPSCs were maintained in mES2i medium, i.e. DMEM supplemented with 15% (v/v) fetal bovine serum, glutamine, non-necessary amino acid, 1000U/ml LIF, 1 μM PD0325901 and 3 μM Chir99021. Our experiments performed with animals were approved by the relevant institutional animal care and use committee (IACUC) of Guangzhou Institutes of Biomedicine and Health (GIBH).

### High-resolution assay of comparative genomic hybridization microarray

Genomic DNAs extracted from each miPSC line and the parental donor (MEF B2) were fragmented using *Alu*I and *Rsa*I enzyme digestion. DNA labeling was conducted using Agilent SureTag DNA Labeling Kit. Different fluorescence dyes were used for DNA labeling of miPSCs (Cy5-dUTP) and the donor parental cell line (Cy3-dUTP). Each labeled miPSC DNA was hybridized together with the labeled donor DNA onto Agilent SurePrint G3 mouse 1 × 1 M microarray for 40 hours at 65**°**C. DNA processing, microarray handling and scanning were conducted following the Agilent oligonucleotide CGH protocol (version 6.0).

### Genome-wide CNV analyses

The microarray scanning profiles were processed by Agilent Feature Extraction 10.7.3.1. The extracted data was analyzed and plotted by Agilent Workbench 7.0. ADM-2 was selected as statistical algorithm with the threshold of 6.0 and the Fuzzy Zero turning on. Each CNV was called by at least four consecutive probes with log_2_Ratio (fluorescence value ratio of miPSC-associated Cy5 to donor-associated Cy3) consistent with deletion or duplication.

### Statistical analysis

The Mann–Whitney *U* test with the exact significance was used to determine statistically significant differences in miPSC CNVs between different reprogramming methods. The ANOVA test was used in Figure 3C when a two-factor test is needed. Differences were considered statistically significant when p-value < 0.05.

## Competing interests

The authors declare that they have no competing interests.

## Authors’ contributions

YC, JC and FZ conceived the study. YC, LG, JC and XZ performed the experiments. LG and XZ identified the iPSC lines. YC, JC, WZ, CZ, JW, LJ, DP and FZ analyzed the data. YC, JC, LJ, DP and FZ draft the manuscript. All authors read and approved the final manuscript.

## Supplementary Material

Additional file 1 Sample information of mouse iPSC lines. Click here for file

Additional file 2 The CNVs identified in mouse iPSC linse. Click here for file

## References

[B1] Takahashi K Yamanaka S Induction of pluripotent stem cells from mouse embryonic and adult fibroblast cultures by defined factors Cell 2006 15 4 663 676 10.1016/j.cell.2006.07.024 16904174

[B2] Robinton DA Daley GQ The promise of induced pluripotent stem cells in research and therapy Nature 2012 15 7381 295 305 10.1038/nature10761 22258608 PMC3652331

[B3] Buganim Y Faddah DA Jaenisch R Mechanisms and models of somatic cell reprogramming Nat Rev Genet 2013 15 6 427 439 10.1038/nrg3473 23681063 PMC4060150

[B4] Yamanaka S Induced pluripotent stem cells: past, present, and future Cell Stem Cell 2012 15 6 678 684 10.1016/j.stem.2012.05.005 22704507

[B5] Ben-David U Benvenisty N The tumorigenicity of human embryonic and induced pluripotent stem cells Nat Rev Cancer 2011 15 4 268 277 10.1038/nrc3034 21390058

[B6] Hussein SM Batada NN Vuoristo S Ching RW Autio R Narva E Ng S Sourour M Hamalainen R Olsson C Copy number variation and selection during reprogramming to pluripotency Nature 2011 15 7336 58 62 10.1038/nature09871 21368824

[B7] Quinlan AR Boland MJ Leibowitz ML Shumilina S Pehrson SM Baldwin KK Hall IM Genome sequencing of mouse induced pluripotent stem cells reveals retroelement stability and infrequent DNA rearrangement during reprogramming Cell Stem Cell 2011 15 4 366 373 10.1016/j.stem.2011.07.018 21982236 PMC3975295

[B8] Ben-David U Benvenisty N High prevalence of evolutionarily conserved and species-specific genomic aberrations in mouse pluripotent stem cells Stem Cells 2012 15 4 612 622 10.1002/stem.1057 22328490

[B9] Mayshar Y Ben-David U Lavon N Biancotti JC Yakir B Clark AT Plath K Lowry WE Benvenisty N Identification and classification of chromosomal aberrations in human induced pluripotent stem cells Cell Stem Cell 2010 15 4 521 531 10.1016/j.stem.2010.07.017 20887957

[B10] Abyzov A Mariani J Palejev D Zhang Y Haney MS Tomasini L Ferrandino AF Rosenberg Belmaker LA Szekely A Wilson M Somatic copy number mosaicism in human skin revealed by induced pluripotent stem cells Nature 2012 15 7429 438 442 10.1038/nature11629 23160490 PMC3532053

[B11] Zhang F Gu W Hurles ME Lupski JR Copy number variation in human health, disease, and evolution Annu Rev Genomics Hum Genet 2009 15 451 481 10.1146/annurev.genom.9.081307.164217 19715442 PMC4472309

[B12] Pinto D Darvishi K Shi X Rajan D Rigler D Fitzgerald T Lionel AC Thiruvahindrapuram B Macdonald JR Mills R Comprehensive assessment of array-based platforms and calling algorithms for detection of copy number variants Nat Biotechnol 2011 15 6 512 520 10.1038/nbt.1852 21552272 PMC3270583

[B13] Tang YC Amon A Gene copy-number alterations: a cost-benefit analysis Cell 2013 15 3 394 405 10.1016/j.cell.2012.11.043 23374337 PMC3641674

[B14] Hanna JH Saha K Jaenisch R Pluripotency and cellular reprogramming: facts, hypotheses, unresolved issues Cell 2010 15 4 508 525 10.1016/j.cell.2010.10.008 21074044 PMC3032267

[B15] Chen J Liu J Chen Y Yang J Chen J Liu H Zhao X Mo K Song H Guo L Rational optimization of reprogramming culture conditions for the generation of induced pluripotent stem cells with ultra-high efficiency and fast kinetics Cell Res 2011 15 6 884 894 10.1038/cr.2011.51 21445094 PMC3203703

[B16] Nakagawa M Koyanagi M Tanabe K Takahashi K Ichisaka T Aoi T Okita K Mochiduki Y Takizawa N Yamanaka S Generation of induced pluripotent stem cells without Myc from mouse and human fibroblasts Nat Biotechnol 2008 15 1 101 106 10.1038/nbt1374 18059259

[B17] Chen J Liu J Yang J Chen Y Chen J Ni S Song H Zeng L Ding K Pei D BMPs functionally replace Klf4 and support efficient reprogramming of mouse fibroblasts by Oct4 alone Cell Res 2011 15 1 205 212 10.1038/cr.2010.172 21135873 PMC3193408

[B18] Wang Y Chen J Hu JL Wei XX Qin D Gao J Zhang L Jiang J Li JS Liu J Reprogramming of mouse and human somatic cells by high-performance engineered factors EMBO Rep 2011 15 4 373 378 10.1038/embor.2011.11 21399616 PMC3077243

[B19] Carey BW Markoulaki S Hanna JH Faddah DA Buganim Y Kim J Ganz K Steine EJ Cassady JP Creyghton MP Reprogramming factor stoichiometry influences the epigenetic state and biological properties of induced pluripotent stem cells Cell Stem Cell 2011 15 6 588 598 10.1016/j.stem.2011.11.003 22136932

[B20] Stadtfeld M Apostolou E Akutsu H Fukuda A Follett P Natesan S Kono T Shioda T Hochedlinger K Aberrant silencing of imprinted genes on chromosome 12qF1 in mouse induced pluripotent stem cells Nature 2010 15 7295 175 181 10.1038/nature09017 20418860 PMC3987905

[B21] Okita K Ichisaka T Yamanaka S Generation of germline-competent induced pluripotent stem cells Nature 2007 15 7151 313 317 10.1038/nature05934 17554338

[B22] Papp B Plath K Reprogramming to pluripotency: stepwise resetting of the epigenetic landscape Cell Res 2011 15 3 486 501 10.1038/cr.2011.28 21321600 PMC3193418

[B23] Polo JM Anderssen E Walsh RM Schwarz BA Nefzger CM Lim SM Borkent M Apostolou E Alaei S Cloutier J A molecular roadmap of reprogramming somatic cells into iPS cells Cell 2012 15 7 1617 1632 10.1016/j.cell.2012.11.039 23260147 PMC3608203

[B24] Esch D Vahokoski J Groves MR Pogenberg V Cojocaru V Vom Bruch H Han D Drexler HC Arauzo-Bravo MJ Ng CK A unique Oct4 interface is crucial for reprogramming to pluripotency Nat Cell Biol 2013 15 3 295 301 10.1038/ncb2680 23376973

[B25] Wang T Chen K Zeng X Yang J Wu Y Shi X Qin B Zeng L Esteban MA Pan G The histone demethylases Jhdm1a/1b enhance somatic cell reprogramming in a vitamin-C-dependent manner Cell Stem Cell 2011 15 6 575 587 10.1016/j.stem.2011.10.005 22100412

[B26] Chen J Liu H Liu J Qi J Wei B Yang J Liang H Chen Y Chen J Wu Y H3K9 methylation is a barrier during somatic cell reprogramming into iPSCs Nat Genet 2013 15 1 34 42 23202127 10.1038/ng.2491

