# Supplementary material for: Genome-wide CNV analysis in mouse induced pluripotent stem cells reveals dosage effect of pluripotent factors on genome integrity
Source: BMC Genomics. 2014 Jan 28;15:79. doi: 10.1186/1471-2164-15-79 (PMC3912500; doi:10.1186/1471-2164-15-79)
Supplement: Additional file 1 — Sample information of mouse iPSC lines. [file 1471-2164-15-79-S1.pdf]

Additional File 1. Sample information of mouse iPSC lines.

Sample Name	Donor Cell	Reprogramming Method			Passage Number
		Vector	Dosage*	Factor	
O_0.5-8	MEF B2	Retrovirus	0.5 ml	O	4
O_0.5-12	MEF B2	Retrovirus	0.5 ml	O	4
O_0.5-14	MEF B2	Retrovirus	0.5 ml	O	4
O_0.5-17	MEF B2	Retrovirus	0.5 ml	O	4
O_0.5-20	MEF B2	Retrovirus	0.5 ml	O	4
O_0.5-23	MEF B2	Retrovirus	0.5 ml	O	4
O_0.5-24	MEF B2	Retrovirus	0.5 ml	O	4
O_0.5-27	MEF B2	Retrovirus	0.5 ml	O	4
O_1.5-7	MEF B2	Retrovirus	1.5 ml	O	4
O_1.5-13	MEF B2	Retrovirus	1.5 ml	O	4
O_1.5-15	MEF B2	Retrovirus	1.5 ml	O	4
O_1.5-16	MEF B2	Retrovirus	1.5 ml	O	4
O_1.5-20	MEF B2	Retrovirus	1.5 ml	O	4
OKS_0.5-1	MEF B2	Retrovirus	0.5 ml	O, K, S	4
OKS_0.5-2	MEF B2	Retrovirus	0.5 ml	O, K, S	4
OKS_0.5-3	MEF B2	Retrovirus	0.5 ml	O, K, S	4
OKS_0.5-4	MEF B2	Retrovirus	0.5 ml	O, K, S	4
OKS_0.5-6	MEF B2	Retrovirus	0.5 ml	O, K, S	4
OKS_0.5-7	MEF B2	Retrovirus	0.5 ml	O, K, S	4
OKS_0.5-9	MEF B2	Retrovirus	0.5 ml	O, K, S	4
OKS_0.5-10	MEF B2	Retrovirus	0.5 ml	O, K, S	4
OKS_0.5-11	MEF B2	Retrovirus	0.5 ml	O, K, S	4
OKS_0.5-20	MEF B2	Retrovirus	0.5 ml	O, K, S	4
OKS_1.5-5	MEF B2	Retrovirus	1.5 ml	O, K, S	4
OKS_1.5-6	MEF B2	Retrovirus	1.5 ml	O, K, S	4
OKS_1.5-9	MEF B2	Retrovirus	1.5 ml	O, K, S	4
OKS_1.5-10	MEF B2	Retrovirus	1.5 ml	O, K, S	4
OKS_1.5-15	MEF B2	Retrovirus	1.5 ml	O, K, S	4
OKS_1.5-16	MEF B2	Retrovirus	1.5 ml	O, K, S	4
OKS_1.5-17	MEF B2	Retrovirus	1.5 ml	O, K, S	4
OKS_1.5-18	MEF B2	Retrovirus	1.5 ml	O, K, S	4
XYZK_0.5-5	MEF B2	Retrovirus	0.5 ml	X, Y, Z, K	4
XYZK_0.5-6	MEF B2	Retrovirus	0.5 ml	X, Y, Z, K	4
XYZK_0.5-7	MEF B2	Retrovirus	0.5 ml	X, Y, Z, K	4
XYZK_0.5-14	MEF B2	Retrovirus	0.5 ml	X, Y, Z, K	4
XYZK_0.5-17	MEF B2	Retrovirus	0.5 ml	X, Y, Z, K	4
XYZK_0.5-19	MEF B2	Retrovirus	0.5 ml	X, Y, Z, K	4
XYZK_0.5-20	MEF B2	Retrovirus	0.5 ml	X, Y, Z, K	4
XYZK_0.5-21	MEF B2	Retrovirus	0.5 ml	X, Y, Z, K	4
XYZK_0.5-26	MEF B2	Retrovirus	0.5 ml	X, Y, Z, K	4
XYZK_0.5-27	MEF B2	Retrovirus	0.5 ml	X, Y, Z, K	4

* MOI=15 when 0.5 ml virus was used.
