# Supplementary material for: Genome-wide CNV analysis in mouse induced pluripotent stem cells reveals dosage effect of pluripotent factors on genome integrity
Source: BMC Genomics. 2014 Jan 28;15:79. doi: 10.1186/1471-2164-15-79 (PMC3912500; doi:10.1186/1471-2164-15-79)
Supplement: Additional file 2 — The CNVs identified in mouse iPSC linse. [file 1471-2164-15-79-S2.pdf]

Additional File 2. The CNVs identified in mouse iPSC lines.

CNV Number	Sample	Chr	Start*	End*	Size (bp)	Gain/Loss
1	O_0.5-12	11	67319120	67416156	97036	Loss
2	O_0.5-14	1	111641112	111698153	57041	Gain
3	O_0.5-14	5	19763889	20055334	291445	Loss
4	O_0.5-14	7	111676075	111694358	18283	Gain
5	O_0.5-17	1	111641112	111698153	57041	Gain
6	O_0.5-17	2	41454989	41514361	59372	Loss
7	O_0.5-17	7	111676075	111694358	18283	Gain
8	O_0.5-17	11	83492037	83498745	6708	Gain
9	O_0.5-17	16	36302510	36320263	17753	Gain
10	O_0.5-20	1	111641112	111698153	57041	Gain
11	O_0.5-20	6	46222174	46813572	591398	Loss
12	O_0.5-20	7	111676075	111694358	18283	Gain
13	O_0.5-20	11	83492037	83498745	6708	Gain
14	O_0.5-23	1	111641112	111698153	57041	Gain
15	O_0.5-23	7	111676075	111694358	18283	Gain
16	O_0.5-23	11	83492037	83498745	6708	Gain
17	O_0.5-24	1	111641112	111698153	57041	Gain
18	O_0.5-24	4	121649423	121786389	136966	Loss
19	O_0.5-24	6	46222174	46822139	599965	Loss
20	O_0.5-24	7	111676075	111694358	18283	Gain
21	O_0.5-24	11	83492037	83498745	6708	Gain
22	O_0.5-24	12	115336085	115357837	21752	Gain
23	O_0.5-24	16	36300878	36319122	18244	Gain
24	O_0.5-24	18	57818889	57900711	81822	Gain
25	OKS_0.5-1	11	83492037	83498745	6708	Gain
26	OKS_0.5-1	X	166409011	166425358	16347	Gain
27	OKS_0.5-3	2	77706709	77848292	141583	Gain
28	OKS_0.5-3	7	111635850	111694358	58508	Gain
29	OKS_0.5-3	9	70334168	70370499	36331	Loss
30	OKS_0.5-3	11	83492037	83498745	6708	Gain
31	OKS_0.5-3	X	166409011	166426594	17583	Gain
32	OKS_0.5-4	7	62806051	63074301	268250	Gain
33	OKS_0.5-6	11	83492037	83498745	6708	Gain
34	OKS_0.5-6	19	5580920	5821987	241067	Gain
35	OKS_0.5-6	X	166409011	166425358	16347	Gain
36	OKS_0.5-9	2	77708141	77842288	134147	Gain
37	OKS_0.5-9	4	111760165	113643468	1883303	Loss
38	OKS_0.5-9	5	62177325	62200468	23143	Loss
39	OKS_0.5-9	7	38959822	38985531	25709	Gain
40	OKS_0.5-9	7	111656562	111694358	37796	Gain
41	OKS_0.5-9	11	83492037	83498745	6708	Gain
42	OKS_0.5-9	14	52575997	52591723	15726	Gain
43	OKS_0.5-9	18	57817169	57900711	83542	Gain
44	OKS_0.5-9	X	166409011	166426594	17583	Gain
45	OKS_0.5-10	11	83492037	83498745	6708	Gain

46	OKS_0.5-10	X	166409011	166426594	17583	Gain
47	OKS_0.5-11	11	83492037	83498745	6708	Gain
48	OKS_0.5-11	X	166409011	166425358	16347	Gain
49	OKS_0.5-20	X	60994919	61154346	159427	Loss
50	OKS_1.5-6	7	111656562	111695908	39346	Gain
51	OKS_1.5-6	11	83492037	83498745	6708	Gain
52	OKS_1.5-6	X	166409011	166428351	19340	Gain
53	OKS_1.5-16	X	166409011	166428351	19340	Gain
54	OKS_1.5-17	18	78430905	78462280	31375	Loss
55	OKS_1.5-17	X	166410871	166423594	12723	Gain
56	OKS_1.5-18	6	87927609	87968595	40986	Gain
57	XYZK_0.5-14	13	15762453	15867460	105007	Loss
58	XYZK_0.5-27	2	77708141	77863815	155674	Gain
59	XYZK_0.5-27	4	121649423	121786389	136966	Loss
60	XYZK_0.5-27	7	38960941	38985531	24590	Gain
61	XYZK_0.5-27	7	111676075	111694358	18283	Gain
62	XYZK_0.5-27	11	83492037	83498745	6708	Gain
63	XYZK_0.5-27	X	166409011	166426594	17583	Gain

*Genome coordinates based on the assembly of NCBI37/mm9 (Mouse July 2007)
